# Viral miRNA delivered by exosomes from Marek's disease virus-transformed lymphoma cell line exerts regulatory function in internalized primary chicken embryo fibroblast cells

**DOI:** 10.1016/j.tvr.2024.200286

**Published:** 2024-06-22

**Authors:** Man Teng, Jun Luo, Yaoyao Zhang, Vishwanatha R.A.P. Reddy, Priya Samuel, Yongxiu Yao, Venugopal Nair

**Affiliations:** a The Pirbright Institute & UK-China Centre of Excellence for Research on Avian Diseases, Pirbright, Guildford, Surrey, United Kingdom; b Institute for Animal Health & UK-China Centre of Excellence for Research on Avian Diseases, Henan Academy of Agricultural Sciences, Zhengzhou, 450002, People's Republic of China; c Henan Provincial Key Laboratory of Animal Immunology, Henan Academy of Agricultural Sciences, Zhengzhou, 450002, People's Republic of China; d Key Laboratory of Animal Immunology, Ministry of Agriculture and Rural Affairs of the People's Republic of China, Zhengzhou, 450002, People's Republic of China; e Department of Biological and Life Sciences, Oxford Brookes University, Oxford, United Kingdom; f Department of Biology, University of Oxford, Oxford, United Kingdom

**Keywords:** MDV, Exosomes, MSB-1, miRNA, Tumorigenesis

## Abstract

In the past decade, research has demonstrated that viral miRNAs encoded by a number of viral genomes, particularly by most of the herpesvirus including Marek's disease virus (MDV), play important regulatory roles in viral infection, replication, and regulation of tumorigenesis. As macrovesicles in cells, exosomes can deliver viral miRNAs and exert gene regulatory functions. Whether the exosomes play a role in the replication, pathogenesis/tumorigenesis of avian herpesviruses such as oncogenic Marek's disease virus (MDV) remains unclear. Herein we extracted and identified the exosomes from MDV-transformed T cell line MSB-1 and demonstrated high abundance of MDV-1 miRNA expression. Using dual luciferase-based reporter assay, we also demonstrated that the exosomes derived from MSB-1 can deliver functional miRNA successfully into primary chicken embryo fibroblasts. These findings provide new insights into the role of exosomes and the mechanisms of how virus-encoded miRNA function in MDV latency/activation switching, viral replication, pathogenesis and/or tumorigenesis.

Marek's disease (MD) caused by Marek's disease virus (MDV) is one of the important avian infectious diseases affecting poultry health. MDV infection causes immunosuppression of chicks, rapid onset T cell lymphoma and high mortality, and is estimated to cause annual economic losses between 1 and 2 billion US dollars globally [1,2]. MDV is a typical α-herpesvirus that undergoes acute cytolytic infection of B cells, activation of T cells, and switch to latent infection followed by T cell transformation which ultimately leads to MD lymphomas and neuronal infiltration [3]. The MDV genome is about 180 kb encoding over 100 open reading frames (ORFs) and a number of non-coding RNAs (ncRNAs), such as microRNAs (miRNAs) and long ncRNAs (lncRNAs) [3]. The function of most of MDV genes in virus life cycle, pathogenesis and tumorigenesis still need further investigation although extensive studies in the last numerous years in several laboratories including ours, have identified some of the major viral proteins and ncRNAs that contribute directly to the neoplastic transformation and development of MD tumors.

miRNAs are a class of small ncRNAs of about 22–24 nucleotides (nt) in length that exert important post-transcriptional regulatory functions in cell development, differentiation, apoptosis, disease progression, as well as tumorigenesis by binding to the coding or the non-coding region of the target gene mRNA transcripts to degrade or inhibit protein translation [4]. In addition to higher eukaryotes, a number of viral genomes, especially those of most herpesviruses, also encode miRNAs and play critical roles in viral replication, infection and pathogenesis [5]. All of the three serotypes of MDV, serotype 1 (MDV-1), serotype 2 (MDV-2) and serotype 3/herpesvirus of Turkeys (HVT), encode tens of miRNAs [6]. In the MDV-1 viral genome, 14 miRNA precursors encode a total of 26 mature molecules and distributed in three gene clusters (Meq-, Mid- and LAT-cluster) [[6], [7], [8]]. The sequences of these miRNAs are highly conserved among different virulence and pathogenic MDV-1 strains. However, these miRNAs encoded by oncogenic MDV-1 strains can be transcribed from the same or independent promoters depending on the stages of MDV infection and pathogenesis [9]. Although the expression levels of these miRNAs can vary, some of the miRNAs from different gene clusters have similar dynamic expression patterns [10,11] indicating that they may have similar regulatory roles.

Recent studies have shown that some of the MDV-1 miRNAs are extremely important in viral replication and tumor induction. For example, deletion of MDV1-miR-M4-5p, a viral ortholog of the host oncogenic miRNA miR-155 [12], can significantly reduce the oncogenicity of the virus [13,14]. MDV1-miR-M4-5p plays an important role in MDV induced oncogenesis by targeting both viral and host genes as well as TGF-beta signaling pathway [15,16]. In addition, MDV1-miR-M3-5p has also been reported to regulate the host SMAD2 gene to provide more space in host cells for viral replication [17]. Another MDV-1 encoded miRNA miR-M7-5p functions at the latent infection stage by targeting immediate-early genes ICP4 and ICP27 [18]. In addition to the progress made in the studies on regulation mechanisms of these MDV-1-encoded miRNAs, we have also found that most of the Meq-clustered miRNAs may play a crucial role in MD pathogenesis/oncogenesis [19] whereas mid-clustered miRNAs may suppress pathogenesis/oncogenesis [20] as demonstrated in animal experiments using mutant viruses generated by the bacterial chromosome clone (BAC) mutagenesis. However, to date, very little is known about the mechanism of these viral miRNA functions.

Exosomes are types of extracellular vesicles with a diameter of about 30–150 nm and have been reported in lower microorganisms and higher eukaryotes [21,22]. Studies have shown that exosomes can carry protein, DNA, mRNA and ncRNA including miRNA, and play important roles in the infection, replication, assembly, and cell communication of several human herpesviruses [[23], [24], [25], [26], [27]]. As viruses and extracellular vesicles (EVs) share biogenesis pathways, the EVs have recently been suggested as a potential source of viral antigens to elicit specific immune responses and develop new vaccination platforms [28]. However, the role of exosomes in the biology of avian herpesviruses has not been fully understood, although the exosomes from serum of MDV-vaccinated and tumor-bearing chicken had been previously isolated and primarily characterized by different approaches [[29], [30], [31]]. To investigate the role of exosomes carrying viral miRNAs in MDV-1 infection and MD tumorigenesis, we isolated exosomes from MDV-induced T lymphoma cell line MSB-1 [32]. Briefly, culture media with exosomes depleted FBS from three T150 flasks of MSB-1 were used for exosomes isolation using size-exclusion chromatography (SEC). The possibility of having virions in the exosome preparation can be excluded due to the latent nature of MDV infection in MDV-transformed cell lines such as MSB-1 where the viral genome is maintained by integration into the host telomeres with limited viral gene expression and no virions present. After the initial centrifugation, filtration, concentration, and fractionation through Sepharose column, 80 μl of purified exosomes was obtained. The concentration of this exosomes prep was determined to be 5.5 E+11 particles/mL. The plotted results of the particle number and protein concentrations (quantified by BCA assay) of each fraction show that the peak of the particle number is distinct from that of the protein concentration in the fractions, showing a good separation using the size exclusion column method. As with mammalian EV preparations, the EVs from chicken cells were primarily collected in fractions 7–9 and efficiently separated from free protein, which eluted in later fractions (Fig. 1A, Top-left panel). The transmission electron microscopy (TEM) images of 1:5000 diluted exosomes prep showed that the extracted MSB-1 exosomes were of high purity and uniform sized with an average diameter of 112.9 nm (Fig. 1A, Top-right panel). Compared with exosomes of other species reported previously which have a homogenous cup-shaped appearance under scanning electron microscopy, the shape of avian exosomes is slightly different. Most of the MSB-1 exosomes particles are smooth-surfaced oval vesicles, which may be species specific (Fig. 1A, Top-right panel). The purified exosomes were also analyzed by nanoparticle tracking analysis using Zetaview particle analyzer (Fig. 1A, lower panel).

**Fig. 1:**
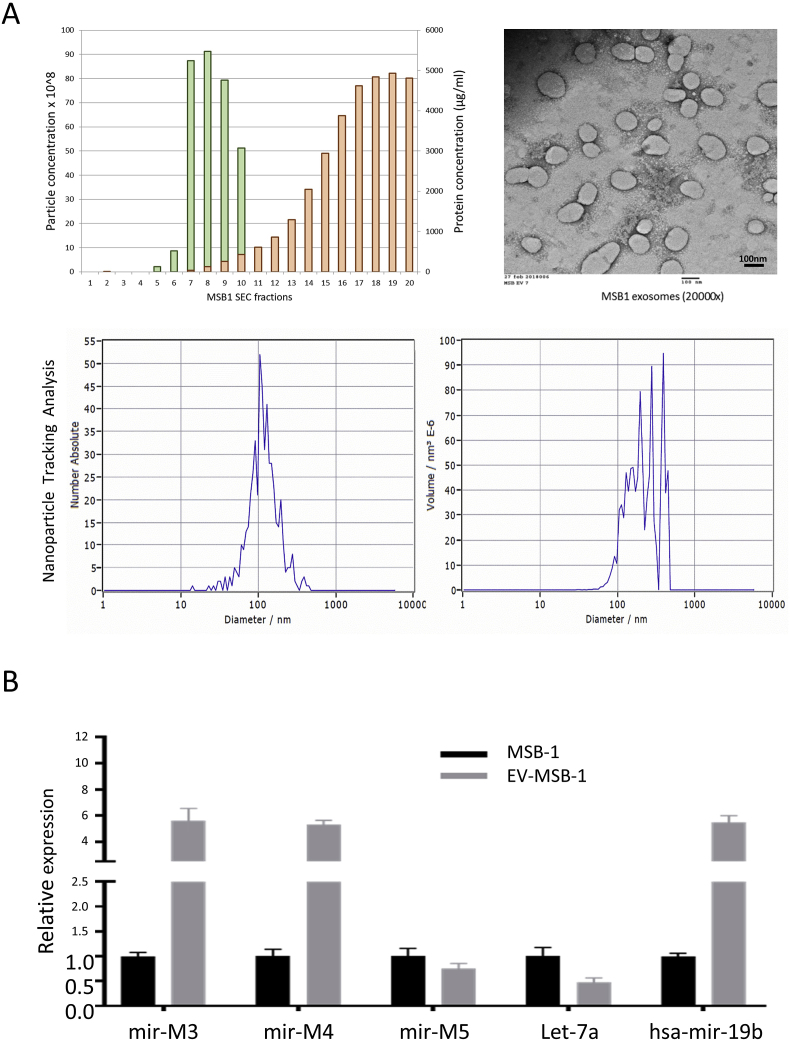
**Characterization of chicken exosomes secreted from the transformed MSB-1 cell line. (A)** The exosomes from MSB-1 cell culture supernatant were purified using SEC and particle number and protein concentration (quantified by BCA assay) are plotted for each fraction of the SEC (A, top-left panel). EVs negatively stained with uranyl acetate are shown on TEM (A, top-right panel). The purified EVs are also analyzed by nanoparticle tracking analysis using Zetaview particle analyser (A, lower panel). **(B)** Expression of miRNAs in chicken exosomes and the corresponding cells detected by RT-qPCR. Total RNAs were extracted from MSB-1 cells and the purified exosomes derived from MSB-1 cells. The expression of representative viral and host miRNAs was determined by RT-qPCR. The data shown is representative of three independent experiments. Error bars indicate triplicate repeats.

Having observed the isolated exosomes from MSB-1 cells using TEM, we next measured the miRNA expression from the isolated exosomes. Total RNA from purified exosomes and MSB-1 cells from which the exosomes derived from was extracted using miRNeasy Mini Kit (QIAGEN), and the expression of selected four viral and one host miRNAs was examined by TaqMan MicroRNA Assay System (Thermo Fisher Scientific, Basingstoke, United Kingdom) as described previously [33]. As expected, the viral miRNAs mdv-1-miR-M3, M4, M5, and host miRNAs gga-let-7a and gga-miR-19b are present in RNA from both MSB-1 and exosomes isolated from it (Fig. 1B). This indicates that exosomes derived from the MDV-induced T lymphoma cell lines MSB-1 specifically carry viral miRNAs.

CD63, a member of the tetraspanin family, highly enriched on the exosomes, are widely used as an exosome marker, along with other biomarkers such as CD9, CD81, CD82, CD53 and CD37 [[34], [35], [36]]. While the human CD63 is well characterized with multiple reagents available for its detection, the less well characterized chicken CD63 homolog lacks specific reagents that can be used for the characterization of the exosome population from chicken cells. To track the MSB-1 cell line-derived exosomes, we first constructed stable MSB-1 cell lines expressing human CD63 tagged with the fluorescent markers. For this, we first transfected the CD63-mScarlet plasmid into MSB-1 and the specific transient expression of the red fluorescently labeled CD63 protein was observed 24 h later (Fig. 2A, top panel). The MSB-1 cells stably expressing CD63-mScarlet was obtained following a serial of passages with puromycin selection and single cell cloning by limiting dilution. As shown in the confocal images (Fig. 2A, bottom panel), the red fluorescence labeled CD63 is expressed in all cells from the expanded population of single MSB-1 clone stably expressing CD63-mScarlet.

**Fig. 2:**
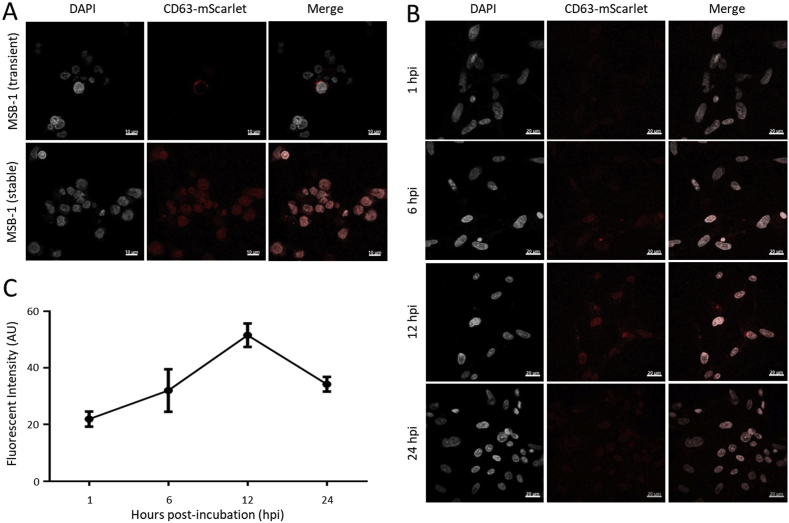
**Expression of the scarlet-CD63 protein in MSB-1 cells and Internalization of scarlet-CD63-labeled MSB-1 exosomes into incubated CEFs. (A)** Transient or stable expression of the scarlet-CD63 protein in MSB-1 cells. **(B)** Internalization of scarlet-CD63-labeled MSB-1 exosomes into incubated CEFs. 100ng/well of the purified exosomes was added into the medium of cultured CEF in 24-well plates and the uptake of exosomes by CEF was visualized with confocal microscope at 1, 6, 12, and 24 h post incubation. **(C)** Quantification of the fluorescence intensity of CEF cells incubated with scarlet-CD63-labeled MSB-1 exosomes at different time points using ImageJ software. The data shown is representative of three independent experiments. Results represent the mean of triplicate assays with error bars showing Standard Error of the Mean.

Exosomes serve as an intercellular communicator. When transferred to recipient cells, its content such as mRNAs and miRNAs remained functional and changed cellular behavior [37]. Following the observation that the exosomes isolated from MSB-1 cells carry viral miRNAs, we wanted to examine whether exosomes can be taken up by another cell type such as chicken embryo fibroblast (CEF) and if the exosomes delivered viral miRNAs are still functional in the acceptor cells. For this, we purified mScarlet-labeled exosomes from MSB-1-CD63-mScarlet cells using SEC method described. The shape and size of these exosomes were identical to the un-labeled exosomes under the TEM (data not shown). 100ng/well of the purified exosomes was added into the medium of cultured CEF in 24-well plates and incubated for the indicated period of time. Following the incubation, the uptake of exosomes by CEF was visualized with confocal microscope. As shown in Fig. 2B, weak red fluorescence was observed in the cytoplasm of CEF at 1 h post incubation (hpi). The intensity of fluorescence in CEF increased significantly at 6 hpi, reached a peak at 12 hpi and decreased at 24 hpi (Fig. 2B). The dynamic changes of the fluorescence level reflecting the exosome uptake by CEF incubated with scarlet-CD63-labeled MSB-1 exosomes at different time points was also quantified using ImageJ software (National Institutes of Health, USA) (Fig. 2C). The data demonstrates that MSB-1 exosomes were internalized and gradually up-taken by co-cultured CEF cells.

The uptake of exosomes by CEF is not only visualized by confocal microscope but also demonstrated by expression of viral miRNAs detected by RT-qPCR. The duplicate samples of MSB-1 exosomes incubated CEFs above were collected and the total RNA was extracted using Qiagen miRNeasy kit. The expression levels of MDV-1 miRNAs, miR-M3, miR-M4 and miR-M5, were detected by TaqMan MicroRNA Assay System. As shown in Fig. 3A, all three viral miRNAs were detected in exosome-incubated CEFs, and the dynamic changes of expression level is consistent with exosomes uptake level shown in confocal images of exosomes internalization (Fig. 2B). This confirms that MSB-1 exosomes can deliver viral miRNAs into primary CEF cells. The fact that the dynamic changes of the expression level of all three MDV-1 miRNAs are in the same trend, further confirming the exosome miRNAs were indeed up-taken by the CEF cells.

**Fig. 3:**
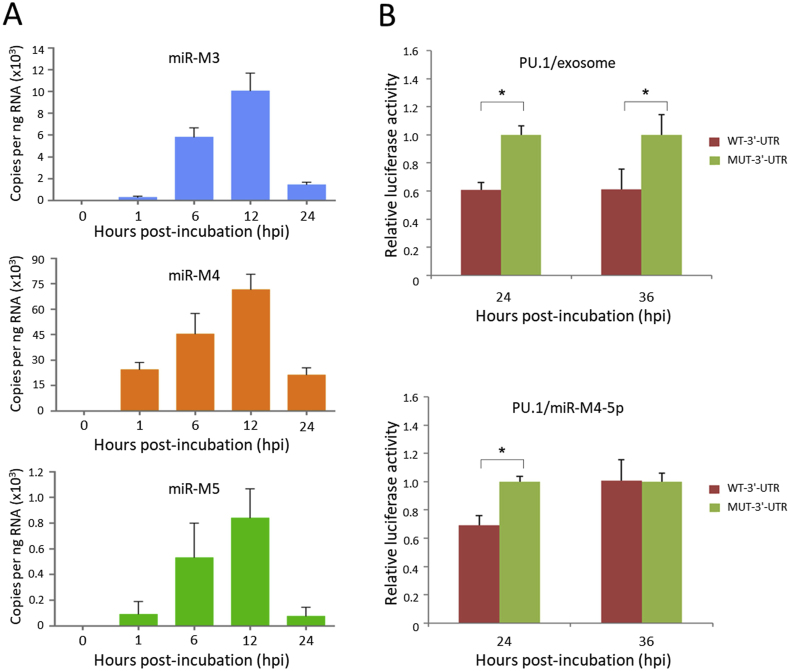
**Exosomes delivered viral miRNA are functional in recipient cells. (A)** Detection of viral miRNAs in MSB-1 exosome-incubated CEF cells. Total RNAs were extracted from MSB-1 exosome-incubated CEF cells collected at different time points post-incubation and used for determining the viral miRNA expression levels by RT-qPCR. Error bars indicate triplicate repeats. **(B)** Confirmation of the interactions between the miR-M4-5p mimics or exosome-delivered miR-M4-5p and its host target PU.1 gene by dual luciferase reporter assay (DLRA). CEF cells were first transfected with psi-CHECK2-PU.1-3′UTR plasmid followed by the transfection of miR-M4-5p mimics or the incubation with MSB-1 exosomes. At 24 or 36 hpi, the cells were treated for determining the ratio of Renilla luciferase/Firefly luciferase activities. The synthetic miR-M4-5p mimic serves as a positive control. WT, wild type; MUT, mutant. The data shown is representative of three independent experiments. Error bars indicate triplicate repeats. The statistical significance of differences was determined by Student t-test, *Significant difference (p < 0.05).

Our previous work had demonstrated that Pu.1 is one of the host target genes of miR-M4-5p [12]. Thus, we used psiCHECK-2 luciferase-based reporter system (Promega) to investigate whether exosome-delivered viral miRNAs are still functional in the recipient cells. Considering the dynamic changes of miRNAs being delivered into CEF cells (Fig. 2, Fig. 3A), we first transfected CEFs with the reporter constructs, psiCHECK2-PU.1-3′-UTR containing Pu.1 3′-UTR with miR-M4-5p binding site or psiCHECK2-Pu.1-3′-UTR-Mut with mutated miR-M4-5p targeting site. Twelve hours later, purified MSB-1 exosomes were added into the culture media of transfected CEF and incubated for 24 and 36 h respectively, followed by analyzing the luciferase activities using Dual-Glo Luciferase Assay System (Promega). The synthesized miR-M4-5p mimic was used as positive control. As shown in Fig. 3B, miR-M4-5p mimics reduced the luciferase level by 40 % in CEF at both time points, whereas MSB-1 exosomes inhibited luciferase expression by 35 % at 24 hpi and no detectable inhibition was observed at 36 hpi. This result indicates that exosome-delivered viral miRNA is functional in the recipient CEF cells. Compared to the synthetic miR-M4-5p mimics, the loss of miRNA function in exosomes incubated cells at 36 hpi is likely due to decreased exosome miRNA level in CEF, which is consistent with the dynamic changes of miRNA level detected by RT-qPCR (Fig. 3A). All the data were acquired from three independent repeats and were statistically calculated as means (M) ± standard deviations (SD) utilizing the software GraphPad Prism Version 6.0 (GraphPad Software, Inc., San Diego, CA, USA). The statistical significance of differences was determined by Student t-test.

Previous studies have shown that the expression levels of most viral miRNAs in MD lymphoma cell lines such as MSB-1 are much higher than those of virus-infected cells [8], which may be closely related to MDV latent infection, cell transformation and subsequent tumorigenesis. Although the MDV genome is in latent state in the tumor cell line such as MSB-1, the virus can be reactivated by co-culturing MSB-1 with CEF. More interestingly, MD tumors can be induced once MSB-1 cells were inoculated into host birds [38]. Although it is not clear whether the tumor formation is due to the direct expansion and growth of inoculated MSB-1 or is being induced by the reactivated virus particles from MSB-1 cells, it is most likely that exosomes are involved in this important process.

Previous studies have shown that exosomes can deliver viral miRNAs as well as DNA, mRNAs, proteins and other ncRNAs [[23], [24], [25], [26], [27]]. In this study, we have extracted and purified the exosomes from the MDV-transformed tumor cell line MSB-1 and confirmed that it can deliver MDV-1 miRNAs that remained functional in recipient cells. Future studies on the transcriptomics and proteomics of these exosomes, together with those from serum exosomes of vaccinated/protected or lymphoma-bearing chickens [31], could provide further clues on the role of exosomes delivered miRNAs in the virally-induced MD pathogenesis/oncogenesis.

## Funding

This project was supported by the 10.13039/501100012166National Key Research and Development Program of China (Grant No. 2023YFE0106100), the 10.13039/501100000268Biotechnology and Biological Sciences Research Council (BBSRC) (grants BBS/E/I/00007032, BBS/E/I/00007038, BBS/E/I/00007039, BBS/E/PI/23NB0003, BB/R007896/1, BBS/E/PI/230002A and BBS/OS/NW/000007), the 10.13039/501100001809National Natural Science Foundation of China (Grant No. U21A20260), and the Natural Science Foundation for Distinguished Young Scholars of Henan Province (Grant No. 232300421009).

## CRediT authorship contribution statement

**Man Teng:** Writing – original draft, Validation, Methodology, Investigation, Funding acquisition, Formal analysis, Conceptualization. **Jun Luo:** Writing – review & editing, Writing – original draft, Validation, Methodology, Investigation, Funding acquisition, Formal analysis, Conceptualization. **Yaoyao Zhang:** Validation, Methodology, Investigation, Formal analysis, Conceptualization. **Vishwanatha R.A.P. Reddy:** Methodology, Investigation. **Priya Samuel:** Methodology, Investigation, Formal analysis. **Yongxiu Yao:** Writing – review & editing, Supervision, Funding acquisition, Formal analysis, Conceptualization. **Venugopal Nair:** Writing – review & editing, Funding acquisition, Formal analysis, Conceptualization.

## Declaration of competing interest

The authors declare that they have no known competing financial interests or personal relationships that could have appeared to influence the work reported in this paper.

## Data Availability

Data will be made available on request.
